# Enhancement of Local Crowd Location and Count: Multiscale Counting Guided by Head RGB-Mask

**DOI:** 10.1155/2022/5708807

**Published:** 2022-08-24

**Authors:** Guoyin Ren, Xiaoqi Lu, Jingyu Wang, Yuhao Li

**Affiliations:** ^1^School of Mechanical Engineering, Inner Mongolia University of Science & Technology, Baotou 014010, China; ^2^School of Information Engineering, Inner Mongolia University of Science & Technology, Baotou 014010, China; ^3^Inner Mongolia University of Technology, Hohhot 010051, China

## Abstract

**Background:**

In crowded crowd images, traditional detection models often have the problems of inaccurate multiscale target count and low recall rate.

**Methods:**

In order to solve the above two problems, this paper proposes an MLP-CNN model, which combined with FPN feature pyramid can fuse the feature map of low-resolution and high-resolution semantic information with less computation and can effectively solve the problem of inaccurate head count of multiscale people. MLP-CNN “mid-term” fusion model can effectively fuse the features of RGB head image and RGB-Mask image. With the help of head RGB-Mask annotation and adaptive Gaussian kernel regression, the enhanced density map can be generated, which can effectively solve the problem of low recall of head detection.

**Results:**

MLP-CNN model was applied in ShanghaiTech and UCF_ CC_ 50 and UCF-QNRF. The test results show that the error of the method proposed in this paper has been significantly improved, and the recall rate can reach 79.91%.

**Conclusion:**

MLP-CNN model not only improves the accuracy of population counting in density map regression, but also improves the detection rate of multiscale population head targets.

## 1. Introduction

At present, image-based crowd counting still faces many problems: (1) Problems such as image clutter, uneven crowd distribution, crowd overlap, and occlusion lead to low head detection rates. (2) Pedestrians have different scales in the image. Due to the difference in the distance between the head and the camera, the head has different scales, so the head with small scale is not easy to be detected. All these reasons have created huge challenges for the further advancement of crowd counting [1–5].

Current crowd counting methods can be divided into two categories: methods based on object detection and feature regression [6–9]. Early work is to use some kind of object detection model to detect individual objects. However, the detection architecture requires a lot of computational resources and cannot better solve the occlusion problem and size feature extraction. When the head is small or occluded, it usually cannot be detected. Therefore, the main problem is the low recall rate of the head. In real dense crowd scenes, small heads are common. As a result, detection-based dense crowd counting tends to be gradually replaced by other methods due to underestimation [10–12].

In the past, head detection can only detect the size of a crowd of dozens of people. When the size of the crowd exceeds a few hundred people, the detection model is difficult to cope with due to the small size and serious occlusion. In contrast, the regression method based on the density map can more reliably obtain the overall characteristics of the crowd and can effectively estimate the number of the crowd [9, 13, 14].

**Figure 1:**
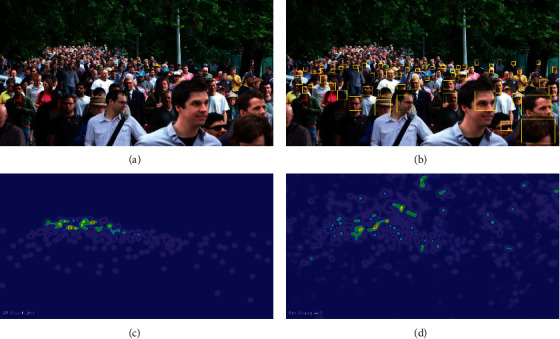
Traditional method density map and detection results. (a) Input image. (b) YOLO V4 crowd head detection results. (c) MCNN ground truth density map. (d) MCNN estimated density.

Usually, the Gaussian kernel is generated with each head as the center, but it does not match the size of the head, and the density map is obviously interfered by the background [15–17]. Therefore, the density map thus generated also suffers from significant deficiencies. As shown in Figure 1, GT and ES are the real density maps and estimated density maps generated by the MCNN model on the ShanghaiTech PartA dataset, and the density maps estimated by MCNN are obviously distorted.

The problem is that using density map regression can only estimate the number but cannot locate the head position, which severely limits the application of crowd counting in video anomaly detection and pedestrian reidentification. As shown in Figure 1, the head detection of the YOLO V4 model cannot detect small-scale heads. In contrast, the RGB human head annotation box provides more information about head localization. If these head ROI pictures can be used as training masks, it will help strengthen the head features and facilitate the estimation of human head size. There are currently methods that utilize adaptive Gaussian kernels to generate high-quality density maps [18, 19]. High-quality density maps train more robust regression networks, providing prior knowledge for crowd detection that is closer to the actual distribution of crowds [20]. One of the reasons that previous detection methods cannot detect small heads is due to the lack of scale perceptron or the limitation of its own structure. For those tiny heads, efficient scale-adaptive perceptrons should be designed. Fortunately, RGB image and head RGB-Mask image feature fusion can provide a prior for estimating head size, which helps to set suitable scale fusion perceptrons for different scales of human heads [21, 22].

Aiming at the shortcomings of the above methods, this paper attempts to use the prior information provided by the density map combined with the RGB-Mask labeled data to achieve a high recall rate and high robustness based on the density map guided detection.

The contributions of our work are summarized as follows:

- In the past, there was not much work to count and detect people of different sizes by using multifeature fusion. In particular, previous work has rarely fused the RGB-Mask feature into the RGB feature. This paper proposes a fusion scheme of “medium-term fusion” between the RGB-Mask feature and the RGB feature. The selection of “medium-term fusion” can not only ensure the effective fusion of the head RGB-Mask feature and the head RGB feature, but also ensure that the head RGB-Mask strengthens the role of local small target features in the vgg16 small target feature extraction process. Therefore, this part of the enhanced small target head feature can be effectively connected with the low-resolution semantic features in the subsequent FPN feature pyramid.
- Through the analysis of previous work, it is found that the traditional FPN feature pyramid starts with high-resolution semantic features, so there are insufficient low-resolution semantic feature information and low-resolution semantic feature map. The improved FPN model starts with low-resolution semantic features and ends with low-resolution semantic features after being fused with high-resolution semantic information features. In this way, the feature map of low-resolution and high-resolution semantic information can be fused with less computation. It can take into account the high semantic features with less information containing small targets and the low semantic features with more information containing small targets. Finally, the feature layer of high semantic content is sampled up and stacked down to ensure the characteristics and information of small targets.
- Through the analysis of previous work, it is found that there are many methods to realize population detection by using cross entropy loss or *L*1 and *L*2 loss functions alone. However, there is less literature on the mixed use of cross entropy loss and *L*1 and *L*2 loss functions. Because the cross entropy loss is only effective for low-density pedestrian detection, it is not suitable for dense crowd detection. Therefore, this paper attempts to combine cross entropy loss with *L*1 and *L*2 loss functions and then realize small-scale head detection with density map regression as the guiding model.

## 2. Related Work

### 2.1. Detection-Based Counting

Early work on crowd counting problems focused on detection counting methods. These works count the total number of pedestrians by detecting body, head, or shoulders [23–27]. Reference [23] proposed a method based on skeleton detection to count the total number of pedestrians in crowd scenes. Specifically, the skeleton map is obtained by foreground segmentation, and the moving target is detected by comparing the difference between the skeleton and the background. The work [24, 25] used a real-time skeleton detection model using OpenPose to detect pedestrians. This method has achieved initial success in sparse populations. However, in the case of occlusion, the detection of multiple human skeletons is abnormal due to overlapping, which will lead to the problem of wrong counts. However, occlusions are common in real-world scenarios, so most pedestrian detection and counting systems fail. To achieve efficient detection, head region-based detection is an effective way to avoid occlusion [26, 27].

In recent years, CNN-based head-and-shoulders pedestrian detection has been fully developed. For example, RCNN [28], Fast RCNN [29], R–FCN [30], or Mask-RCNN [31] can be applied in low-density crowd counting, but these detection models are not very good in small object detection. The reason is that these models are not designed with an effective head scale processing strategy to deal with small target objects. For another group of methods such as Overfeat [32], YOLO [33], or SSD [34], although these frameworks can detect some objects with smaller scales, the detection performance is poor, especially in small objects with large detection errors. Although SSD has a good performance in balancing computation time and accuracy, the above methods are obviously unable to cope with dense crowds with serious occlusion because no effective strategy is designed.

Crowd counting is an extremely challenging job. Currently crowded images are divided into two categories: crowded images that can be resolved and small-resolution clumps that cannot be resolved. For discriminable crowded crowds, crowd counting can be done using regression-based methods. Much literature [35–39] uses regression methods to implement the crowd counting problem. These methods first extract local edge features and texture features of crowd images and then learn a regression function to estimate the sum of all local counts in the image. A regression function is used to build a mapping from local features to counts. Commonly used regression functions include linear regression [35], piecewise linear regression [36], ridge regression [37], Gaussian process regression [38], and neural networks [39].

A small head in an indistinguishable crowd image only covers 10 to 20 pixels, so there is not enough information to extract pedestrian features; Ji et al. [40] consider the difficulty of learning such features and therefore use random forest regression to learn the nonlinear mapping between local patch features and density maps. Following this work, Sadler et al. [41] used random forests to regress crowd density, and the training efficiency was also greatly improved. In [42], Mo mentioned a response of a Laws filter convolved with mask to obtain a two-dimensional density layer and finally realized the regression of difficult-to-distinguish crowd images, where mask is to create a mask by the gray-scale restricted area growth method. In other words, methods based on these regressions are more likely to fail in crowd counting in image areas with high crowd density due to the lack of deeper features. Therefore, the counting problem of visually indistinguishable crowded images cannot be completely solved.

### 2.2. Density Map Regression

Methods based on regression density maps have achieved a breakthrough in addressing indistinguishable crowd counting [8, 43–49]. Powerful CNNs play an important role in the density map regression process, and Wang et al. [43] show that features extracted from deep models are more effective than handcrafted features. Compared with the regression-based method, the density map regression-based method preserves a large amount of spatial distribution information in the crowd area, so the density map regression is more suitable for analyzing small targets. The crowd counting process is to first regress the density map of the crowd and then get the count by integrating the density map.

Pai [44] et al. aim to achieve dense crowd counting in visually indistinguishable crowded images. This method convolves image patches with a Gabor filter and classifies the responses of the Gabor filter with a support vector machine (SVM). This method is effective for counting both high-density crowd images and low-density crowd images in a specific scene, but the counting effect of replacing it with other scenes cannot take effect, and the migration performance is not good.

In reference [44] proposed density map regression with an adaptive Gaussian kernel, which can better handle density map estimation in regions with different density levels. Miangoleh et al. [45] attempted to learn various density levels to integrate contextual information and generate high-resolution density maps. Reference [45] also proposed to use density map regression results to guide detection. Reference [46] proposed a framework called Hydra-CNN, which achieves the final density prediction by extracting a pyramid of image feature blocks at multiple scales. Zhang et al. adopted a CNN with geometric or perspective information to fuse scale-dependent contextual information to achieve multiscale perception. Zhang et al. [48] fused features from different counting network layers to obtain robust representations for scale changes. Reference [49] proposed a Deep Scale Purification Network (DSPNet) to extract multiscale features and compensate for the loss of context. Sam et al. [50] proposed Switch CNN, which trains an optimal regressor for a specific input, thereby improving the counting ability.

Density map regression based on deep learning [51–53] has solved many dense crowd counting problems in the past few years, but it also has some shortcomings: although it can increase the location information of crowds in crowded images, it cannot locate pedestrians border. This limits further applications in surveillance domains such as pedestrian tracking and reidentification tasks.

### 2.3. Density Map Regression Guided Detection

In order to simultaneously estimate the number of human heads and detect bounding boxes when regressing the density map, Zhong et al. [54] used the density map regression to improve the head detection results. But that method does not work for cross-scene counting. The research most relevant to this paper is Hou et al.'s [55] using cross-modal data to achieve crowd counting in RGB-D images with the help of a regression guided detection network (RDNet). Leverage density maps improve head detection rates in detection networks. To improve the robustness of the method, the detector directly classifies anchors into specific classes and regresses bounding boxes in a dense manner. These convolutional features usually only capture basic visual patterns and lack strong semantic information, which may lead to many false positive results.

## 3. Methods

The overall architecture of the method described in this paper includes two kernels, the head RGB-Mask head perceptron and the adaptive Gaussian kernel density map regressor. This section will deeply analyze the internal mechanism of this method from the perspective of formula principle and structure. The head RGB-Mask perceptron is implemented with the help of MLP-CNN network. The regression guided detection of the adaptive Gaussian density map is realized with the help of the MR-CNN network. The training data in this paper uses the head RGB-Mask binarization and the mask head annotation box as the input to strengthen the head supervision training.

### 3.1. Adaptive Gaussian Kernel Density Map

Adaptive Gaussian kernel regression is able to produce density maps that are closer to the true density map. The adaptive Gaussian kernel can gradually approach the guide size of the head mask through training. The density maps produced with the help of regression can provide prior knowledge for the head detection module of the MR-CNN network. This prior can guide the location and size of the generated head detection boxes.

#### 3.1.1. Gaussian Density Map

Crowd estimation requires the conversion of labeled head images into crowd density maps. Assuming that an image has *N* heads, its original formula is expressed as

(1) $$\begin{array}{c} H\left(x\right)=\sum_{\text{i}=1}^{N}\delta \left(x-x_{\text{i}}\right). \end{array}$$

*δ* is the impulse function, *x*_*i*_ is the position of the head in the pixel, *δ(x−x*_*i*_) is the impulse response function of the head position in the image, and *N* is the total number of heads in the image. The density map based on the traditional Gaussian kernel can be expressed as

(2) $$\begin{array}{c} F\left(x\right)=\sum_{i=1}^{N}\delta \left(x-x_{i}\right)*G_{\sigma_{i}}\left(x\right),\\ \sigma_{i}=\beta S_{i}. \end{array}$$

Among them, *S*_*i*_ in formula (2) is the average distance of the nearest *m* heads from the head of *x*_*i*_. In formula (2), *S*_*i*_ is approximately equal to the size of the head in a dense crowd. Here the experimental parameter *β* is adaptively adjusted according to the actual crowding degree of each image. The size of the Gaussian kernel is variable.

#### 3.1.2. Head RGB-Mask Adaptive Estimation

In order to make the density map better correspond to the images of different head sizes of dense crowds, the traditional Gaussian kernel function is improved and a Gaussian kernel based on head RGB-Mask geometric adaptability is proposed.

On the basis of the traditional Gaussian density map formula, the prior knowledge is used to further enhance the adaptability of the Gaussian kernel to the head RGB-Mask features. Different from the prior knowledge of previous algorithms, this paper proposes a new head RGB-Mask perception prior knowledge, which further highlights the target of the head RGB-Mask prior by considering the position and size relationship of the head RGB-Mask geometric constraints. This prior knowledge is represented by a Gaussian model as

(3) $$\begin{array}{c} G\left(x,y,d^{i}\right)=\text{exp}\left[-\left(\frac{{\left(x-\mu_{x}\right)}^{2}}{2\sigma_{x}^{2}}+\frac{{\left(x-\mu_{y}\right)}^{2}}{2\sigma_{y}^{2}}+\frac{{\left(x-\mu_{d^{i}}\right)}^{2}}{2\sigma_{d^{i}}^{2}}\right)\right]. \end{array}$$

Among them, *µ* indicates the position of the Gaussian peak; *σ* controls the shape of the Gaussian curve; the smaller *σ* is, the steeper the curve is; (*x*^*θ*^, *y*^*θ*^, *d*^i^) is the coordinate of the pixel *θ* in the normalized image coordinate system. The *XY* plane corresponds to the image plane, and *d i* corresponds to the head RGB-Mask size of the image.

The density map regression module takes an image as input and utilizes a CNN for density map estimation. The density map generation strategy is to use the head RGB-Mask adaptive Gaussian kernel to generate the density map. Given a training set of heads with annotated boxes, if the image contains a total of *N* heads, the adaptive Gaussian kernel density map of the image can be written as

(4) $$\begin{array}{c} F\left(x\right)=\sum_{i=1}^{N}\delta \left(x-x_{i}\right)*G\left(x,y,d^{i}\right). \end{array}$$

*G(x, y, d* ^*i*^ *)* is a 2D Gaussian kernel with adaptive bandwidth, thus transforming the crowd counting problem into the following problem: *F* : *I(x)*⟶*F(x)*, which learns from the image space *I(x)* to the density map space *F(x)* mapping. When the mapping function *F(x)* is established, a density map for any given image can be obtained, and the integral over the entire image is an estimate of the total head count.

### 3.2. Head RGB-Mask Perception Network

*RGB-Mask Perceptron.* For the head RGB-Mask perceptron, the annotated head RGB-Mask dataset was used to train MLP-CNN. MLP-CNN includes multiple scalable submodules, and each submodule unit consists of a VGG16 network. In order to find a reasonable structure of the MLP-CNN variant, here each VGG16 unit of the MLP-CNN is connected in series and parallel. Among them, the RGB-Mask features are captured by the first ten convolutional layers of VGG16. Finally, the RGB-Mask information features of MLP-CNN will enter the RGB feature network from the mid-end entry and finally complete the feature fusion of head RGB and head RGB-Mask.

**Figure 2:**
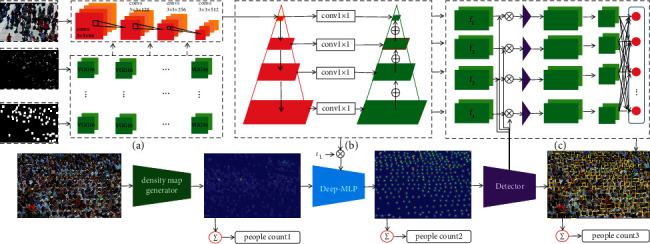
Overview of the proposed network. (a) Each input image is first processed by MLP-CNN. (b) The extracted features are fed to FPN for obtaining representations with spatial context information from different depths of the network and predicting density maps. (c) The spatial context representation is sent to MR-CNN to detect the final crowd.

*RGB feature network.* The RGB network model contains 4 convolutional layers (convs1-convs4). Conv1 has 32 7 × 7 × 64 filters, conv2 has 32 7 × 7 × 128 filters, conv3 has 32 7 × 7 × 256 filters, and the last convolutional layer has 64 5 × 5 × 512 filters device. The convolutional layer uses a max-pooling layer with a kernel size of 2 × 2. Fully connected layers (fc5, fc6, and fc7 not shown in Figure 2) rapidly reduce the spatial resolution.

*The Head RGB-Mask and RGB Fusion Network.* As shown in Figure 2(a), different entrances are used to fuse the head RGB-Mask and RGB model. The head RGB and head RGB-Mask inputs can be directly concatenated, resulting in a new first convolutional layer. It is called early fusion. The scores of the head RGB network and head RGB-Mask branch can also be concatenated at the end of the network and then use 1 × 1 convolution as the classifier. It is called late fusion.

*This Paper Adopts Mid-Term Fusion.* Although early fusion is more expressive than mid-level fusion, it can fully exploit the correlation between features. However, the larger the amount of data expressing the power, the higher the required training cost. The benefit of late fusion is that most of the network initialization weights can be reused directly without readjusting the network weights based on additional inputs. Unfortunately, it does not allow the network to learn about such high-level interdependencies between individual input modalities, since only the resulting scores at the classification level are fused.

Finally, the scores of the head RGB-Mask branch can be merged before a max-pooling layer of the RGB network followed by a 1 × 1 convolutional layer. The number of MLP-CNN modules used in this mid-level fusion method is determined by the desired spatial dimension in the RGB network. Therefore, these models realize the optimal design according to the number of VGG16 of MLP-CNN module, taking into account the training cost and the high-level interdependence between various input modes.

*FPN (Feature Pyramid Network).* In order to achieve multiscale target processing, a feature pyramid structure is added here, as shown in Figure 2(b). The purpose of using feature pyramid is to increase the processing power of CNN for head scale transformation.

The model on the left side of the feature pyramid is called bottom-up. The network first performs the traditional bottom-up top-down feature convolution (left side of the figure), and then the feature map on the left side of the FPN fuses adjacent feature maps from top to bottom. The model on the right is called top-down, and the horizontal arrows are called lateral connections. The purpose of this is that the high-level feature semantics is more, and the low-level feature semantics is less but with relatively more location information.

The specific method is that the higher-level features of the two feature layers use the interpolation method to complete the 2-fold upsampling; that is, on the basis of the original image pixels, the interpolation algorithm is used to insert new pixels between the pixels, and the feature size is doubled. The lower-level features are changed by 1 × 1 convolution to change the number of channels of the lower-level feature, and then the corresponding elements of the result after upsampling and 1 × 1 convolution are simply added. The horizontal connection should use 1 × 1 convolution to change the number of channels, so that the channels of each level processing result are 256-d, which is convenient for classifying the added features later.

With the improved FPN network structure, head RGB-Mask annotation is used as a priori under feature training, and head RGB-Mask plays a role in strengthening local small target features in the VGG16 small target feature extraction process. Therefore, this part of the strengthened small target head features can be effectively connected with low-resolution semantic features. Starting from the low-resolution semantic features, after fusion with the high-resolution semantic information features, it ends with the low-resolution semantic features. It can fuse the feature map with strong low-resolution semantic information and the feature map with weak high-resolution semantic information but rich spatial information under the premise of less computation. The improved FPN network can take into account the high semantic features with less information containing small targets and the low semantic features with more information containing small targets. Finally, the feature layer of high semantic content is sampled up and stacked down to ensure the features and information of small targets.

*Density Map Generator.* First, the frame coordinates of the human head in the original image should be calibrated, and the density function should be obtained with the help of the Gaussian kernel function. However this assumes that each Gaussian kernel is independent in the sample space. In fact, head pixels are inconsistent in scale in different distance regions due to scale variation. Also, in practice, it is impossible to obtain the size of the head accurately due to the occlusion of the human head, so it is difficult to find the relationship between the size of the head and the density map. Therefore, in the same scale area, the average distance of adjacent heads is used as a parameter, so the difference between the generated density map and the real density map is large, as shown in Figure 2.

*RGB-Mask Perceptron.* In order to accurately estimate the population density, it is necessary to consider adding the head RGB-Mask perception parameter to the adaptive Gaussian kernel function. Due to the consideration of image distortion, usually the geometry of the head cannot be determined in the original scene, because the original image lacks the spatial constraint information of the head pixels. In order to obtain the spatial constraint information of the head pixels, the perceptron fused with the head RGB-Mask image information is used as the head range constraint information. Human heads of different scales can give the reasonable range of the head RGB-Mask for the geometrically distorted part. The parameter *σ* of the adaptive Gaussian kernel is determined for each head size.

*MR-CNN Detector.*The detection network takes the features of human heads of different scales as input. Estimate the center point of each scale head object. Then, the head mask reinforcement learning is used to close the head center point to the reinforcement feature boundary and finally represent them with detection boxes, as shown in Figure 2(c).

## 4. Experiments

### 4.1. Dataset Selection Training Configuration

#### 4.1.1. Dataset Introduction and Evaluation Criteria

The crowd counting method in this paper has been evaluated experimentally on three standard datasets, ShanghaiTech, UCF_CC_50, and UCF-QNRF, as shown in Table 1. ShanghaiTech contains part_A_Final and part_A_Final two parts; this paper uses three datasets for model training and testing. The feasibility and applicability of our proposed method are verified by experimental comparison. This paper first gives the relevant parameters of the three datasets used in the experiments. Then, the comparison results between the method used in this paper and the current state-of-the-art crowd counting methods under these datasets are given, and the crowd detection results with high recall rate are given. Finally, this paper conducts ablation experimental studies to demonstrate the independent effectiveness of each method unit in our comprehensive approach.

*Metrics.* Mean absolute error (MAE), mean squared error (RMSE), and cross entropy are used to evaluate crowd counting work. MAE loss is also known as *L*1 loss; RMSE loss is also known as *L*2 loss:

(5) $$\begin{array}{c} \text{MAE}=\frac{1}{N}\sum_{1}^{N}\left|N_{i}-n_{i}\right|,\\ \text{RMSE}=\sqrt{\frac{1}{N}\sum_{1}^{N}{\left(N_{i}-n_{i}\right)}^{2}}. \end{array}$$

*N* is the total number of test images, *N*_*i*_ is the actual number of people in the *i*th test image, and *n*_*i*_ is the estimated number of people in the *i*th image.

(6) $$\begin{array}{c} L=\frac{1}{N}\sum_{i}L_{i}\\ =\frac{1}{N}\sum_{i}-\left[y_{i}\log\left(p_{i}\right)+\left(1-y_{i}\right)\cdot \;\log\left(1-p_{i}\right)\right]. \end{array}$$

*y *_i_ represents the label of sample *i*. Head class is 1, nonhead class is 0. *p*_*i*_ represents the probability that sample *i* is predicted to be head class.

#### 4.1.2. Advantages of Cross Entropy Loss Combined with *L*1 and *L*2 Loss

MAE and RMSE generally depend on the assumption of Gaussian distribution. Therefore, *L*1 and *L*2 loss are more suitable for regression problems. Therefore, the common regression density map method can better complete the counting of dense population, but it is difficult to meet the problem of dense population detection at the same time. Because *L*1 and *L*2 loss cannot be applied to dense crowd images with non-Gaussian distribution under the classification task, the detection effect will be very poor, and small-scale head can not be detected. Cross entropy does not rely on the assumption of Gaussian distribution. Therefore, the combination of cross enterprise in classification detection can make up for the problem that *L*1 and *L*2 loss cannot be fully detected in dense population distribution. Another reason is that, relative to *L*1 and *L*2 loss, the cross entropy loss is monotonic as a whole. The greater the loss, the greater the gradient. It is convenient for gradient descent backpropagation and optimization. Therefore, for classification problems, cross entropy is often used as loss function.

Since the model of this paper is a technical route of density map regression guided detection, from the perspective of training, density map regression based on Gaussian distribution is the primary task of our work, and the use of head density points in density map is a favorable premise for guided detection. Therefore, our work is to complete the training based on adaptive Gaussian regression model and then complete the head detection based on head enhancement feature learning. Here, *L*1 and *L*2 loss are used for the training of adaptive Gaussian regression model, and cross enterprise completes the training of head detection model on this basis. Therefore, cross entropy combined with *L*1 and *L*2 loss can be competent for the overall training of density map regression guided detection model.

#### 4.1.3. Dataset Parameter Setting and Training

**Figure 3:**
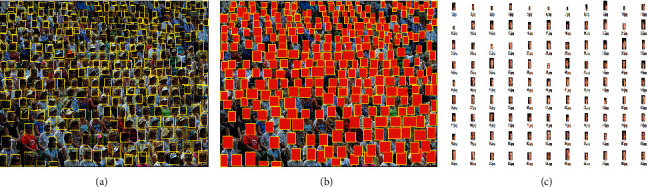
Head RGB-Mask preprocessing process. (a) Original image; (b) RGB-Mask of heads; (c) RGB of heads.

*Preprocessing.* The acquisition of the head RGB-Mask needs to go through two preprocessing steps. The following processes are all implemented by programming, as shown in Figure 3. The rectangular RGB image of the head is cropped by the head annotation frame in the dataset. Pixels outside the head annotation are replaced with RGB-Mask. The RGB image is converted into small head images, which are used to highlight the mask feature of the head and finally convert it into an RGB image.

**Figure 4:**
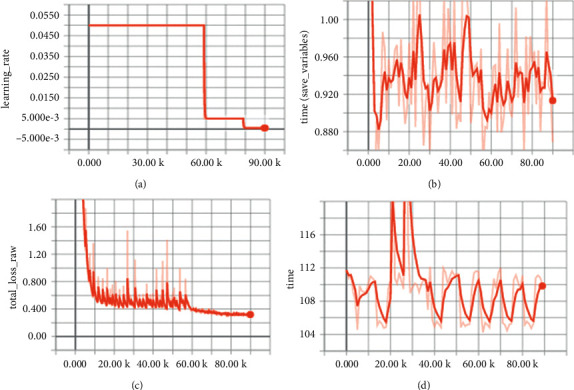
The training process. (a) Learning rate setting curve. (b) Time variation curve of training and saving weights. (c) Loss function variation curve. (d) Total training time variation curve.

*MLP-CNN Training Settings.* MLP-CNN is trained end-to-end. The initial value of Gaussian parameter in MLP-CNN is set to 0.5, and the standard deviation is set to 0.02. In our experiments, MLP-CNN chooses stochastic gradient descent (SGD) with momentum and uses a small learning rate for ShanghaiTech dataset, UCF_CC_50 dataset, and UCF-QNRF dataset to train the model, the initial learning rate is set to 0.005, and the momentum is set to 0.85. After this setting, the training convergence speed is faster, as shown in Figure 4. The implementation of our method is completed under the Pytorch framework. In terms of hardware, three NVIDIA 1080 Ti GPU graphics cards and four Intel(R) Xeon(R) E5-2630 v4 CPU are used to ensure the performance requirements of graphics cards and computing units.

### 4.2. Comparison with State-of-the-Art Methods

#### 4.2.1. Crowd Counting

Experimental data were collected on the state-of-the-art methods in crowd counting from 2015 to 2021, give the performance of these methods on these different datasets, and give the results of the comparison between the methods used in this paper and the current state-of-the-art crowd counting methods. From Table 2, it can be found that the performance of the advanced method gradually improves as the method approaches as the year, so this paper only compares the results of the method closest to ours in 2021, as shown in Table 2.

**Figure 5:**
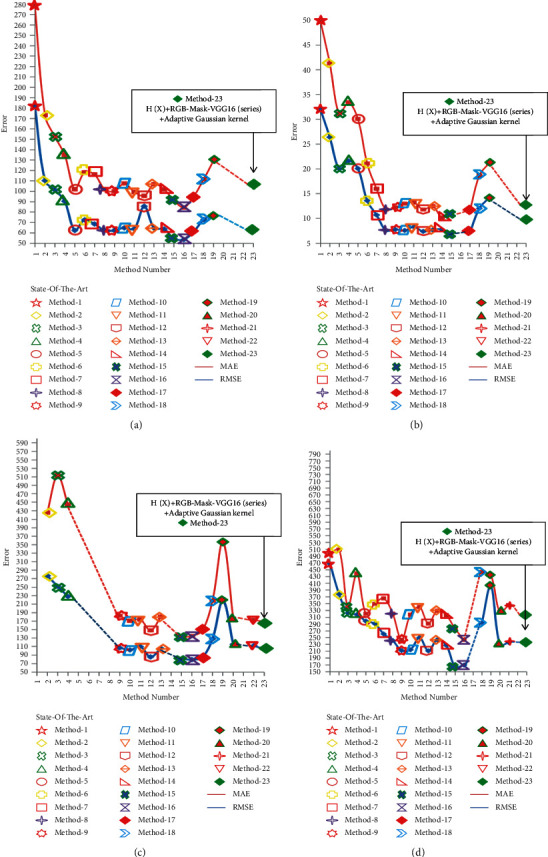
Visualization error results of ShanghaiTech PartA, ShanghaiTech PartB, UCF-QNRF, and UCF_CC_50 datasets. (a) ShanghaiTech PartA error curve; (b) ShanghaiTech PartB error curve; (c) UCF-QNRF error curve; (d) UCF_CC_50 error curve.

**Figure 6:**
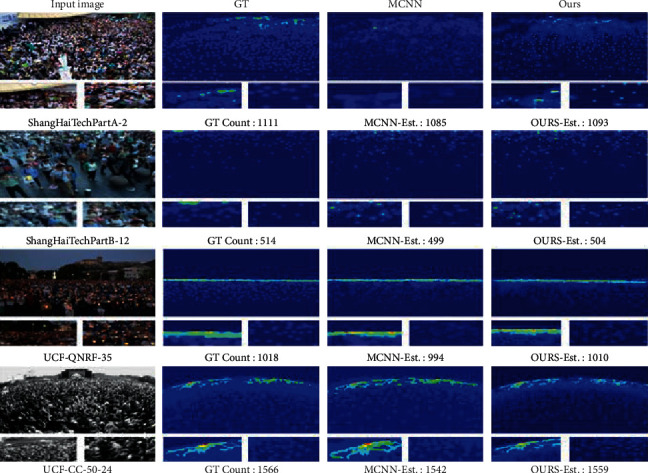
Visualization results of ShanghaiTech PartA, ShanghaiTech PartB, UCF-QNRF, and UCF_CC_50 datasets. From left to right: input image, ground truth density map, MCNN results, and results of our recommended method.

*ShanghaiTech Dataset.* Our method is compared with other state-of-the-art methods on PartA and PartB of the ShanghaiTech dataset. The specific performance is as follows: for PartA on the ShanghaiTech dataset, our results achieve an 8.89/6.01 improvement in MAE and RMSE metrics compared to the state-of-the-art method Partial Annotations in 2021. In particular, our results are 46.3/67.6 better than SFCN in 2019 and 0.9/1.9 better than MCNN in 2016, which is a clear improvement over the PartA count on the ShanghaiTechA dataset, as shown in Figure5(a). For PartB of the ShanghaiTech dataset, our method achieves 2.45/6.11 improvements in MAE and RMSE metrics compared to the state-of-the-art method Partial Annotations in 2021. In particular, our results are 1.12/3.41 better than ic-CNN in 2018 and 16.82/28.71 better than the classic MCNN in 2016, and analyzing the qualitative results shows that our method performs well in databases with different degrees of crowding, as shown in Figure 5(b). At the same time, the density map and the density map of ground truth are more prominent than the crowd Gaussian boundary. Compared with MCNN, the saliency of the human head part is more obvious, as shown in Figure 6.

**Figure 7:**
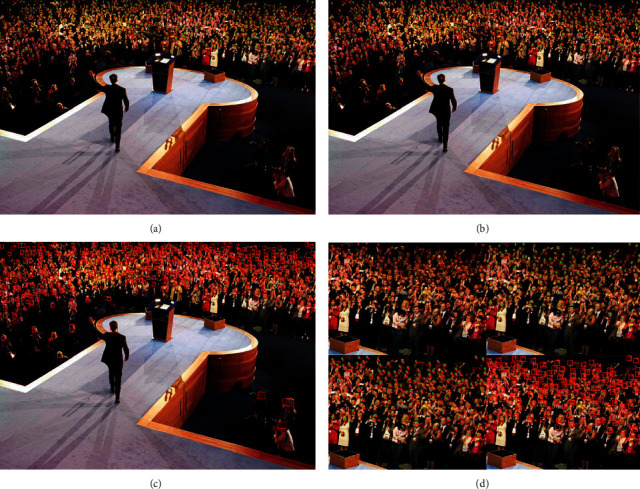
Localization results on the UCF-QNRF dataset. (a) Green points represent ground truth; (b) red points represent estimated positions; (c) detection results; (d) local method renderings.

*UCF-QNRF Dataset.* The performance results of our method on the UCF-QNRF dataset are shown in Table 2. From the results, it is found that our method achieves a 24.52/49.36 improvement in MAE and RMSE metrics compared to the state-of-the-art method Partial Annotations in 2021. In particular, our results are 1.99/11.81 better than DUBNet in 2020 and 173.39/257.31 better than the classic MCNN in 2016. This performance is also a clear improvement in the count of the UCF-QNRF dataset, as shown in Figure 5(c). The density map is compared with the density map of ground truth. The crowd Gaussian boundary is more prominent. Compared with MCNN, the saliency of the human head part is more obvious, as shown in Figure 7.

*UCF_CC_50 Dataset.* The performance results of our method on the UCF_CC_50 dataset are shown in Table 2. From the results, it is found that our method achieves 55.36/125.81 improvement in MAE and RMSE metrics compared to the state-of-the-art method Partial Annotations in 2021. In particular, our results are 5.17/12.02 better than DUBNet in 2020. Our results are 138.97/191.82 better than the classic MCNN in 2016. This performance is also a significant improvement in the count of the UCF_CC_50 dataset, as shown in Figure 5(d). The density map and the ground truth density map are more prominent than the crowd Gaussian boundary. Compared with MCNN, the saliency of the human head part is more obvious, as shown in Figure 7.

Analysis of the overall qualitative results shows that our method performs well in databases of varying degrees of crowding. The main reason is that our proposed network learns more head RGB-Mask spatial context information, which is consistent with our original motivation. The results verify the effectiveness of our method.

The conclusion after comparison is that this method is applied in UCF-QNRF. The performance of UCF-QNRF dataset is better than that of DFN, SS-CNN, and RPNs models. The performance of UCF_CC_50 dataset is better than that of DFN model, but the error performance is worse than that of SS-CNN and SD-CNN models, as shown in Table 3. The reason is that SS-CNN and SD-CNN have made a lot of contributions in the multiscale sensing mechanism, but in the too dense crowd, the method in this paper only uses the improved PFN to judge the head size of small targets which has certain limitations. In addition, in ShanghaiTech dataset, the method error used in this paper is slightly better than DFN.

In addition to the design characteristics of each method, the form of dataset training and annotation will directly affect the counting accuracy of the model for dense populations. Generally, SSL uses labeled and unlabeled data to fit the model, but unlabeled data may make the model worse. FSL performs best because it completely labels all samples, but the labeling cost is too high. Although SSAL can reduce the labeling cost, using some fully labeled images for network training will lose the head posture, illumination, image perspective, and other information of unused labeled images. Pal can maximize the retention of the head posture, illumination, image angle, and other information of the pictures in the dataset, while using less annotation to achieve more accurate full annotation to complete more accurate crowd calculation. Therefore, pal is generally better than SSAL.

#### 4.2.2. Model Complexity and Processing Time Experiment

At UCF_QNRF dataset, this method compares the most advanced counting networks in terms of model parameters (Params) and processing time (Time/s) in order to verify the model's complexity and time consumption. Model parameters (Params) are used to measure the complexity of the model, and processing time (Time/s) is used to measure the time-consuming performance of the model. Through comparison, it is found that the method described in this paper adds FPN and fusion mechanism to the model, so there are many parameters. However, too many model parameters increase the image processing time, so some time-consuming performance is sacrificed. For mlp-cnn, Params = 14.25 × 10^6^, and Time = 2.39 s, as shown in Table 3.

#### 4.2.3. Crowd Detection

At present, the head detection of dense crowds cannot be detected according to the human head scale, and most detection methods are powerless for small pixel heads. The estimation and detection of head position points are particularly important in reflecting the distribution of the crowd. The dense crowd dataset gives the coordinates of the center point of the crowd head annotation box. First, the center point of the head from the real point is marked (the green point is the center point of the rectangular annotation frame), as shown in Figure 7(a). Then the method of this paper extracts the head center point (red point) in the density map, as shown in Figure 7(b). The localization performance of our method on the ShanghaiTech dataset is evaluated by evaluating the precision and recall between the extracted estimated location points (red points) and ground truth annotated head center points (green points), as shown in Figure 7(c).

**Figure 8:**
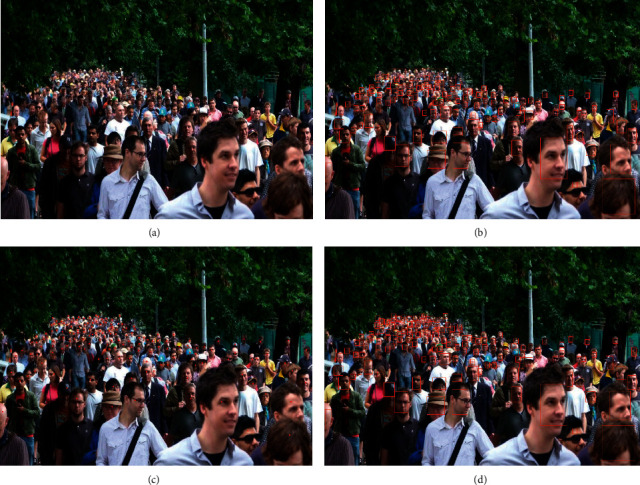
Cross entropy loss improves the detection rate of dense crowds. (a) Input image; (b) detection result of ours method; (c) location point estimation; (d) detection result of ours method.

Before using cross entropy loss, our method has the problem of missing detection in detecting small-scale human heads, as shown in Figure 8(b). There are various crowd scale of the estimated location points, as shown in Figure 8(c). With the help of the cross entropy loss, heads with different scales can be well detected, especially small heads. The positioning result is shown in Figure 8(d). Compared with current more sophisticated feature extraction detection frameworks, the method results in outperforming other methods in terms of precision and recall. This is because the spatial context information of the head RGB-Mask image can constrain the size range of the adaptive Gaussian kernel. In density map head classification, cross entropy can avoid the decline of learning rate of mean square error loss function, the assumption of Gaussian distribution, and the gradient explosion problem caused by *L*1 and *L*2, which can effectively improve the validity of the detection results.

### 4.3. Ablation Study

#### 4.3.1. Effectiveness of the Head RGB-Mask Adaptive Gaussian Kernel

In this part, the ablation experiment is carried out on the RGB-Mask adaptive Gaussian kernel. As shown in Table 4, four different variables were selected for qualitative analysis; namely, the Gaussian kernel function *G*(*X*), the density function *H*(*X*), the multivariate Gaussian function *G*(*X*_*n*_), the difference of the head RGB-Mask perceptron combinations are evaluated. From the results, it can be seen that the density function *H*(*X*) using the Gaussian kernel function *G*(*X*) has a large error in the counting result. It is worth noting that the density function *H*(*X*) of *G*(*X*) does not converge. The reason is that *G*(*X*) cannot obtain the boundary constraints of head spatial context information from different dimensions and is not suitable for the convergence of denser crowds. The degree function *H*(*X*) using the multivariate Gaussian kernel function *G*(*X*_n_) is more suitable for the parallel processing of crowd counting results in terms of counting results, and the processing time is shortened. Therefore, the introduction of the head RGB-Mask perceptron can constrain the edge expansion of each Gaussian kernel, and the convergence time is shortened. This means that the combination of multivariate Gaussian kernel function *G*(*X*_n_) with perception of head RGB-Mask information helps crowd counting with smaller MAE and RMSE errors.

This part is the ablation study of variables in MLP-CNN. As shown in Table 4, three different MLP-CNN variants are explored, RGB-Mask-VGG16 is an MLP-CNN variant with only one VGG16, RGB-Mask-VGG16 (series) is an MLP containing two concatenated VGG16-CNN variants, and RGB-Mask-VGG16 (parallel) is an MLP-CNN variant containing two parallel VGG16s. From the results the MLP-CNN variant head RGB-Mask perceptron actually improves the counting results (except that RGB-Mask-VGG16 (series) does not converge). Furthermore, the MLP-CNN variant of RGB-Mask-VGG16 (parallel) is more efficient than using only one VGG16 because the parallel input of two Mask1 and Mask2 in the head RGB-Mask perceptron helps to strengthen the head ROI head characteristics of the region. It is worth noting that RGB-Mask-VGG16 (series) does not converge. The reason is that the Mask1 and Mask2 feature modules of the head RGB-Mask obtain the head RGB-Mask features from the same dimension at the same position, and the spatial context information can effectively learn the difference of the head region. However, the concatenated structure of the head RGB-Mask feature loses the corresponding relationship of this feature, which will lead to ambiguity in the selection of the same feature. Important information of the crowd count RGB-Mask may be lost.

As shown in Table 5, the reason for choosing VGG16 as encoders: after comprehensively considering a variety of encoders, it is found that VGG16 can effectively improve the processing efficiency of Google inception *V*1, while VGG19 and inception *V*2 and *V*3 models can finally extract more effective features, but too complex network models may bring overfitting and training pressure to training.

#### 4.3.2. Effectiveness of the Head RGB-Mask Feature Fusion Method

This part also discusses how to use the head RGB-Mask information in the adaptive multivariate Gaussian kernel. Four different feature fusion combination schemes are tried, and the results are shown in Table 6. From the results, the feature fusion results using only RGB and head RGB-Mask are not as good as the density map regression using only adaptive Gaussian kernels. This is because there is a certain feature coupling relationship between RGB and head RGB-Mask. However, compared with the adaptive Gaussian kernel, the adaptive Gaussian kernel can reflect the spatial interaction of multihead RGB-Mask features. The feature fusion of RGB and head RGB-Mask can only identify complex channel features. Neither of the individual channels used in combination with the adaptive Gaussian kernel is comparable. The reason is that the coupling degree of local features of channel information or head RGB-Mask feature information is still not optimal. Using the fusion feature of RGB and head RGB-Mask, the head RGB-Mask channel features of the adaptive Gaussian kernel can be mined. Invalid iterations to predict the final crowd density map can be suppressed. Therefore, the combination of adaptive Gaussian kernel and multimodal feature fusion of RGB and head RGB-Mask is the best combination for crowded counting networks.

#### 4.3.3. Effectiveness of Dense Crowd Object Detection Based on Cross Entropy Loss

**Figure 9:**
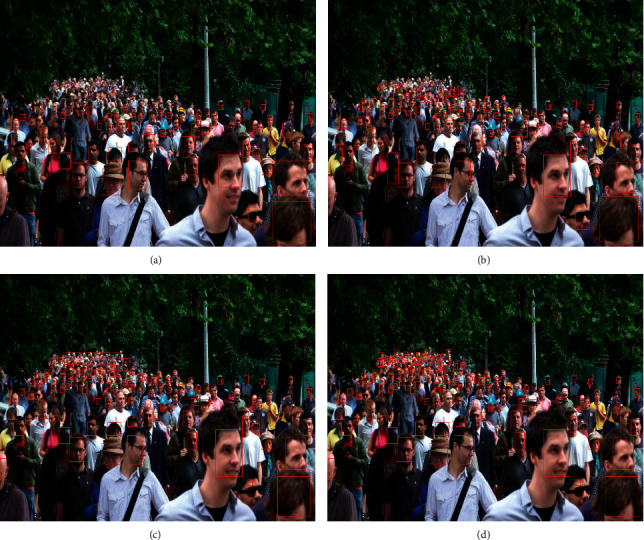
Comparison of detection results of different detection methods. (a) Cross entropy loss only. (b) YOLO V4 test results; (c) YOLO V5 test results; (d) our (*L*1&*L*2) test results.

This method discusses the ablation experiment of the combination of cross entropy loss and *L*1 and *L*2 loss, so as to guide more accurate crowd head detection and complete effective crowd positioning. Therefore, different combination schemes were carried out, and the results are shown in Table 7. As can be seen from the results, the use of cross entropy loss alone makes it impossible to identify crowd with large scale differences, as shown in Figure 9(a). Cross entropy loss is only effective for pedestrian detection with low density and is not suitable for dense crowd detection. From Figures 9(b) and 9(c), YOLO *V*4 and YOLO *V*5 cannot identify people with smaller scales. Therefore, cross entropy loss is necessary to use the density map regression generated after *L*1 and *L*2 loss training as a priori guidance for detection. The combination of cross entropy loss and *L*1 and *L*2 loss can realize small-scale head detection, as shown in Figure 9(d).

**Figure 10:**
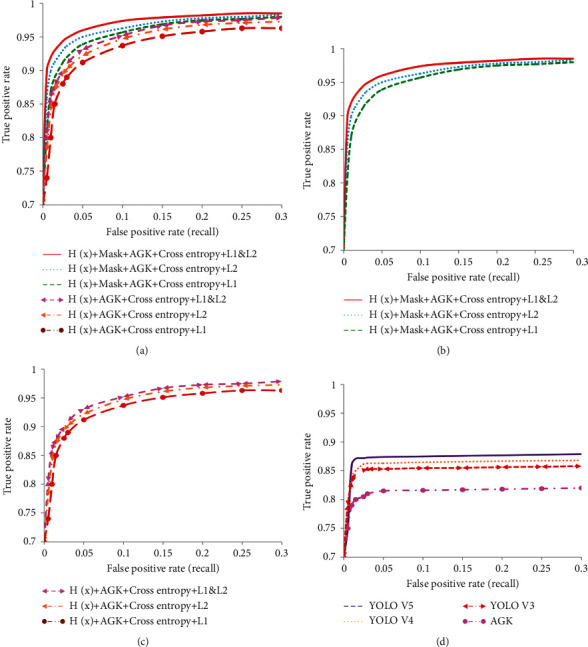
Precision-recall curves for all object classes. (a) Average precision-recall curves of our object classes. (b) Average precision-recall curves with mask. (c) Average precision-recall curves without mask. (d) Average precision-recall curves with Yolo VX detector series and AGK.

In the above cases, the combination of *H*(*x*) + RGB-Mask + AGK + Cross entropy loss + *L*1 and *L*2 loss has the best detection results for people with large density differences. From the comparison of precision-recall curves in all cases in Figures 10(b) and 10(c), it highlights the progressiveness of using the mask method. The combination of cross entropy loss and *L*1 and *L*2 loss method used in this paper has the largest precision and recall rate.  Figure 10(d) shows the analysis of the detection results of four target detection frameworks. No matter which detection framework is used alone, it is not applicable to the detection of dense population. If the head detection of dense population is completed, the help of the combination of *H*(*x*) + RGB-Mask + AGK + cross entropy loss + *L*1 and *L*2 loss is needed in this method.

## 5. Conclusions

In this paper, this method proposes a population counting and detection model. Our MLPNet uses the first ten layers of VGG-16 for feature extraction; our proposed MLP-CNN uses a fusion network based on RGB and head RGB-Mask to extract image channel features and uses an adaptive Gaussian kernel model to extract image spatial edge constraints features and estimates crowd density maps. Cross entropy combined with *L*1 and *L*2 loss functions ensures the accuracy of density map regression guided detection model and improves the results of dense population counting and small head detection. Experiments are conducted on ShanghaiTech dataset, UCF_CC_50 dataset, and UCF-QNRF dataset, and our method achieves equally satisfactory results in crowd counting as other state-of-the-art techniques. Detection network can detect uneven scale, noisy, multidensity crowd. This improves localization performance for smaller populations in the crowd.

**Figure 11:**
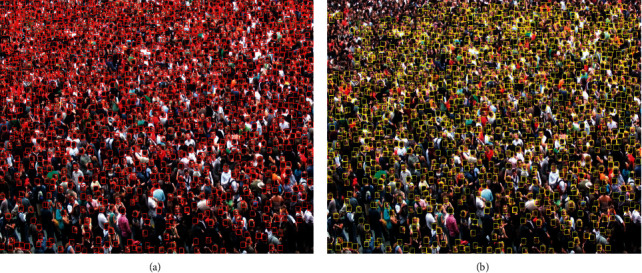
Crowd detection in ShanghaiTech PartA dataset. (a) Ground truth. (b) Real detection.

MLP-CNN has certain limitations in detecting crowd counts in too dense areas. When the crowd scale is too dense and there are too many small-scale heads, there will be large errors in crowd detection and counting. For example, Figure 11 shows the crowd detection results in ShanghaiTech PartA dataset, Figure 11(a) shows the ground truth annotation of the crowd, and Figure 11(b) shows the actual detection results. It can be clearly seen from Figure 11(b) that, in the most crowded part of the crowd, head detection can only detect a small number of heads with obvious characteristics, but the detection rate of heads without obvious characteristics in overcrowded people is very low. In areas with relatively low congestion, the detection rate is very high. Although the PFN scale pyramid and mask fusion module included in the method used in this paper can improve the detection accuracy of some small-scale heads, when the crowd is too dense, the occlusion problem of high-density people is serious, the head resolution is low, and the head features are confused. Therefore, in practical application, this method is largely limited by congestion, resolution, and occlusion. These problems need to be solved in the future.

## 6. Discussion

The comparison of visualization results also demonstrates the effectiveness of our method for crowd detection in complex scenes. In the future, we will extend our approach to video crowd counting and detection, in particular, the effectiveness of the algorithm in improving the overall real-time processing power.

## Figures and Tables

**Table 1:** Summarizations of crowd counting datasets for evaluation.

Dataset	Part	Resolution	Number of images	Max	Min	Avg	Total
ShanghaiTech	PartA	Different	482	3139	33	501.4	241677
	PartB	768 × 1024	716	578	9	123.6	88488
UCF-QNRF	All	Different	1535	12895	49	815.4	1251642
UCF_CC_50	All	Different	50	4543	94	1279.5	63974

**Table 2:** Comparison of the different state-of-the-art methods on ShanghaiTech (SHA&SHB), UCF-QNRF (UQF), and UCF_CC_50 (U50) dataset.

Method		Year	MAE (SHA)	RMSE (SHA)	MAE (SHB)	RMSE (SHB)	MAE (UQF)	RMSE (UQF)	MAE (U50)	RMSE (U50)
1	Crowd CNN [56]	FSL	181.8	277.7	32	49.8	∗	∗	467	498.5
2	MCNN [57]	FSL	110.2	173.2	26.4	41.3	277	426	377.6	509.1
3	CMTL [58]	SSAL	101.3	152.4	20	31.1	252	514	322.8	341.4
4	Switch CNN [50]	FSL	90.4	135	21.6	33.4	228	445	318.1	439.2
5	CP-CNN [59]	FSL	62.4	102	20.1	30.1	∗	∗	298.8	320.9
6	IG-CNN [56]	FSL	72.5	118.2	13.6	21.1	∗	∗	291.4	349.4
7	ic-CNN [60]	FSL	68.5	116.2	10.7	16	∗	∗	260.9	365.5
8	PACNN [61]	FSL	62.4	102	7.6	11.8	∗	∗	241.7	320.7
9	CAN [62]	FSL	62.3	100	7.8	12.2	107	183	212.2	243.7
10	SFCN [63]	USL	64.8	107.5	7.6	13	102	171	214.2	318.2
11	ANF [64]	FSL	63.9	99.4	8.3	13.2	110	174	250.2	340
12	DM-count [65]	FSL	85.6	95.7	7.4	11.8	85.6	148.3	211	291.5
13	DUBNet [66]	FSL	64.6	106.8	7.7	12.5	105.6	180.5	243.8	329.3
14	SDANet [67]	FSL	63.6	101.8	7.8	10.2	∗	∗	227.6	316.4
15	UEPNet [68]	FSL	54.64	91.15	6.38	10.88	81.13	131.68	165.24	275.9
16	SDNET [69]	SSL	53.6	84.4	∗	∗	79.2	134.8	169.4	243.6
17	Gen.loss [70]	SSAL	61.3	95.4	7.3	11.7	84.3	147.5	∗	∗
18	P.Annotations [71]	PAL	72.79	111.61	12.03	18.7	128.13	218.05	293.99	443.09
19	DFN [72]	SSAL	77.58	129.7	14.1	21.10	218.2	357.4	402.3	434.1
20	SS-CNN [73]	FSL	—	—	—	—	115.2	175.7	229.4	325.6
21	SD-CNN [74]	FSL	—	—	—	—	—	—	235.7	345.6
22	RPNs [10]	FSL	—	—	—	—	112	173	—	—
23	Ours	SSAL	63.9	105.6	9.58	12.59	103.61	168.69	238.63	317.28

Statement: USL (Unsupervised Learning), FSL (Full Supervised Learning), SSL (Semisupervised Learning), SSAL (Semisupervised Active Learning), PAL (Partial Annotations Learning).

**Table 3:** Detailed information comparison of the error, complexity, and time consumption of the state-of-art on the UCSD dataset.

Method	MAE (UCF_QNRF)	RMSE (UCF_QNRF)	Params	Times (s)
MCNN [57]	277	426	0.13 × 10^6^	0.02
CMTL [58]	252	514	2.68 × 10^6^	0.45
Switch CNN [50]	228	445	1.543 × 10^6^	0.25
CAN [62]	107	183	4.68 × 10^6^	0.78
SFCN [63]	102	171	5.87 × 10^6^	0.98
ANF [64]	110	174	4.67 × 10^6^	0.78
DM-count [65]	85.6	148.3	16.28 × 10^6^	2.73
DUBNet [66]	105.6	180.5	5.69 × 10^6^	0.95
UEPNet [68]	81.13	131.68	4.68 × 10^6^	0.78
SDNET [69]	79.2	134.8	13.25 × 10^6^	2.22
Gen.loss [70]	84.3	147.5	10.17 × 10^6^	1.70
P.Annotations [71]	128.13	218.05	8.89 × 10^6^	1.49
RPNs [10]	112	173	2.15 × 10^6^	0.36
Ours	103.61	168.69	14.25 × 10^6^	2.39

**Table 4:** Effectiveness analysis of different RGB-Mask model combinations (*C*.1∼*C*.6) in PartA of ShanghaiTech University; √ is choice, and × is not choice.

Component	*C*.1	*C*.2	*C*.3	*C*.4	*C*.5	*C*.6	Ours
*G*(*X*)	√	×	√	×	×	×	—
*H*(*X*)	√	√	√	√	√	√	—
*G*(*X*_n_)	×	√	×	√	√	√	—
RGB-Mask-VGG16	×	×	√	√	×	×	—
RGB-Mask-VGG16 (parallel)	×	×	×	×	√	×	—
RGB-Mask-VGG16 (series)	×	×	×	×	×	√	—
MAE	—	115.9	88.3	79.9	72.5	—	63.9
RMSE	—	186.6	138.8	124.9	116.7	—	105.6

**Table 5:** Comparison between VGG16 and more complex encoder results (*C*.7∼*C*.11); √ is choice, and × is not choice.

Component	*C*.7	*C*.8	*C*.9	***C***.10	*C*.11
RGB-Mask-VGG16	√	×	×	×	×
RGB-Mask-VGG19	×	√	×	×	×
RGB-Mask-inception *V*1	×	×	√	×	×
RGB-Mask-inception *V*2	×	×	×	√	×
RGB-Mask-inception *V*3	×	×	×	×	√
MAE	63.8	63.1	63.9	62.8	55.9
RMSE	105.5	104.9	105.6	103.1	98.5

**Table 6:** Different feature fusion (*C*.1∼*C*.4) and normalization methods on ShanghaiTech PartA; √ is choice, and × is not choice.

Component	*C*.1	*C*.2	*C*.3	*C*.4	Ours
RGB	√	×	√	√	—
RGB-Mask	×	√	√	√	—
Adaptive Gaussian kernel	√	√	×	√	—
MAE	111.6	83.79	79.6	72.7	63.9
RMSE	175.6	136.61	125.9	116.2	105.6

**Table 7:** Comparison of different classification detection results on ShanghaiTechA dataset; √ is choice, and × is not choice.

Component	*C*.1	*C*.2	YOLO *V*4	YOLO *V*5	Ours (&*L*1)	Ours (&*L*2)	Ours (*L*1&*L*2)
*L*1 loss	×	√	—	—	√	—	√
*L*2 loss	×	√	—	—	—	√	√
Cross entropy	√	×	—	—	√	√	√
Precision (%)	80.29	95.62	86.51	87.79	95.58	96.23	97.75
Recall (%)	71.39	75.48	72.68	73.74	75.59	76.98	79.91

## Data Availability

The ShanghaiTech dataset, UCF_CC_50 dataset, and UCF-QNRF dataset used to support the findings of this study are available from the corresponding author upon request.

## References

[1] Sindagi V. A., Patel V. M. (2018). A survey of recent advances in CNN-based single image crowd counting and density estimation. *Pattern Recognition Letters*.

[2] Bai H., Chan S. H. G. (2020). CNN-based single image crowd counting: network design, loss function and supervisory signal.

[3] Qiu Z., Liu L., Li G., Qing W., Nong X., Liang L. Crowd Counting via Multi-View Scale Aggregation networks.

[4] Khan S. D. (2019). Congestion detection in pedestrian crowds using oscillation in motion trajectories. *Engineering Applications of Artificial Intelligence*.

[5] Li J., Wang Y., Wang C., Tai Y., Jianjun Q., Jian Y. Dsfd: dual shot face detector.

[6] Pang J., Li C., Shi J., Zhihai X., Huajun F. (2019). R2-cnn: Fast tiny object detection in large-scale remote sensing images. *IEEE Transactions on Geoscience and Remote Sensing*.

[7] Tian Z., Shen C., Chen H., Tong H. (2019). Fcos: fully convolutional one-stage object detection. https://arxiv.org/abs/1904.01355.

[8] Gao Y., Yang H. Crowd Counting via Multi-Level Regression with Latent Gaussian Maps.

[9] Liu C., Huang Y., Mu Y., Xiaoming Y. (2021). DRENet: Giving Full Scope to Detection and Regression-Based Estimation for Video Crowd Counting. *International Conference On Artificial Neural Networks*.

[10] Khan S. D., Basalamah S. (2021). Scale and density invariant head detection deep model for crowd counting in pedestrian crowds. *The Visual Computer*.

[11] Liu J., Gao C., Meng D., Alexander G. Decidenet: counting varying density crowds through attention guided detection and density estimation. *Proceedings of the IEEE Conference on Computer Vision and Pattern Recognition*.

[12] Ma T., Ji Q., Li N. (2018). Scene invariant crowd counting using multi‐scales head detection in video surveillance. *IET Image Processing*.

[13] Shi Y., Sang J., Tan J., Zhongyuan W., Bin C., Nong S. (2021). *GC-MRNet: Gated Cascade Multi-Stage Regression Network for Crowd Counting*.

[14] Teoh S. K., Yap V. V., Nisar H. Fast Regression Convolutional Neural Network for Visual Crowd Counting.

[15] Li B., Huang H., Zhang A., Liu P., Liu C. (2021). Approaches on crowd counting and density estimation: a review. *Pattern Analysis and Applications*.

[16] Liu X., Yang J., Ding W., Tieqiang W., Zhijin W., Junjun X. (2020). *Adaptive Mixture Regression Network with Local Counting Map for Crowd counting*.

[17] Lian D., Li J., Zheng J., Weixin L., Shenghua G. Density map regression guided detection network for rgb-d crowd counting and localization.

[18] Wan J., Chan A. (2019). Adaptive density map generation for crowd counting. *Proceedings of the IEEE/CVF International Conference on Computer Vision*.

[19] Madani H., Kooshafar M., Emadi M. (2020). Compressive strength prediction of nanosilica-incorporated cement mixtures using adaptive neuro-fuzzy inference system and artificial neural network models. *Practice Periodical on Structural Design and Construction*.

[20] Zhang S., Li H., Kong W. (2021). A cross-modal fusion based approach with scale-aware deep representation for RGB-D crowd counting and density estimation. *Expert Systems with Applications*.

[21] Jiang S., Lu X., Lei Y., Liu L (2020). Mask-Aware networks for crowd counting. *IEEE Transactions on Circuits and Systems for Video Technology*.

[22] Yao H. Y., Wan W. G., Li X. (2020). Mask guided gan for density estimation and crowd counting. *IEEE Access*.

[23] Olivero J. A. T., Anillo C. M. B., Barrios J. P. G., Montoya M., Julianan G., Zamora d. Comparing State-Of-The-Art Methods of Detection and Tracking People on Security Cameras video.

[24] Wang S., Wang Y., Wang X., Xin Y., Huaiming L., Xuelong C. (2019). *An Improved Two-Stage Multi-Person Pose Estimation Model*.

[25] Suzuki S., Amemiya Y., Sato M. Enhancement of gross-motor action recognition for children by CNN with OpenPose.

[26] Zhang L., Shi M., Chen Q. Crowd Counting via Scale-Adaptive Convolutional Neural network.

[27] Shi M., Yang Z., Xu C., Qijun C. Revisiting Perspective Information for Efficient Crowd counting.

[28] Cai Z., Vasconcelos N. Cascade R-Cnn: Delving into High Quality Object detection.

[29] Girshick R. Fast r-cnn.

[30] Dai J., Li Y., He K. (2016). Object detection via region-based fully convolutional networks. *Advances in Neural Information Processing Systems*.

[31] He K., Gkioxari G., Dollár P. Mask r-cnn.

[32] Sermanet P., Eigen D., Zhang X., Michael M., Rob F., Yann L. (2020). *Overfeat: Integrated Recognition, Localization and Detection Using Convolutional networks*.

[33] Laroca R., Severo E., Zanlorensi L. A. A Robust Real-Time Automatic License Plate Recognition Based on the YOLO detector.

[34] Liu W., Anguelov D., Erhan D. (2016). *Ssd: Single Shot Multibox detector*.

[35] Ryan D., Denman S., Sridharan S., Fookes C. (2015). An evaluation of crowd counting methods, features and regression models. *Computer Vision and Image Understanding*.

[36] Yang L., Liu S., Tsoka S., Papageorgiou L. G. (2016). Mathematical programming for piecewise linear regression analysis. *Expert Systems with Applications*.

[37] Saleh A. K. M. E., Arashi M., Kibria B. M. G. (2019). *Theory of ridge Regression Estimation with applications*.

[38] Schulz E., Speekenbrink M., Krause A. (2018). A tutorial on Gaussian process regression: m. *Journal of Mathematical Psychology*.

[39] Albawi S., Mohammed T. A., Al-Zawi S. Understanding of a Convolutional Neural network.

[40] Ji Q., Zhu T., Bao D. (2020). A hybrid model of convolutional neural networks and deep regression forests for crowd counting. *Applied Intelligence*.

[41] Sadler J. M., Goodall J. L., Morsy M. M., Spencer K. (2018). Modeling urban coastal flood severity from crowd-sourced flood reports using Poisson regression and Random Forest. *Journal of Hydrology*.

[42] Mo H., Ren W., Xiong Y. (2020). Background noise filtering and distribution dividing for crowd counting. *IEEE Transactions on Image Processing*.

[43] Wang Y., Hou J., Chau L. P. Object Counting in Video Surveillance Using Multi-Scale Density Map regression.

[44] Pai A. K., Karunakar A. K., Raghavendra U. A Novel Crowd Density Estimation Technique Using Local Binary Pattern and Gabor features.

[45] Miangoleh S. M. H., Dille S., Mai L., Sylvain P., Yagiz A. Boosting monocular depth estimation models to high-resolution via content-adaptive multi-resolution merging.

[46] Liu Y., Shi M., Zhao Q., Xiaofang W. Point in, box out: beyond counting persons in crowds.

[47] Chu H., Tang J., Hu H. (2021). Attention guided feature pyramid network for crowd counting. *Journal of Visual Communication and Image Representation*.

[48] Zhang B., Wang N., Zhao Z., Abraham A., Liu H. (2021). Crowd counting based on attention-guided multi-scale fusion networks. *Neurocomputing*.

[49] Zeng X., Wu Y., Hu S., Wang R., Ye Y. (2020). DSPNet: deep scale purifier network for dense crowd counting. *Expert Systems with Applications*.

[50] Sam Babu D., Surya S., Venkatesh Babu R. Switching Convolutional Neural Network for Crowd counting.

[51] Sam D. B., Peri S. V., Sundararaman M. N., Amogh K., Venkatesh B. (2020). Locate, size, and count: accurately resolving people in dense crowds via detection. *IEEE Transactions on Pattern Analysis and Machine Intelligence*.

[52] Kang D., Ma Z., Chan A. B. (2019). Beyond counting: comparisons of density maps for crowd analysis tasks—counting, detection, and tracking. *IEEE Transactions on Circuits and Systems for Video Technology*.

[53] Liu Y., Shi M., Zhao Q., Xiaofang W. Point in, box out: beyond counting persons in crowds.

[54] Zhong Z., Li J., Zhang Z. An Attention-Guided Deep Regression Model for Landmark Detection in cephalograms.

[55] Hou F., Lei W., Li S., Xi J., Xu M., Luo J. (2021). Improved Mask R-CNN with distance guided intersection over union for GPR signature detection and segmentation. *Automation in Construction*.

[56] Sam D. B., Sajjan N. N., Babu R. V., Mukundhan S. Divide and grow: capturing huge diversity in crowd images with incrementally growing cnn.

[57] Zhang Y., Zhou D., Chen S., Shenghua G., Yi M. Single-image crowd counting via multi-column convolutional neural network.

[58] Wang J., Gao Y., Züfle A., Jingyuan Y. Incomplete Label Uncertainty Estimation for Petition Victory Prediction with Dynamic features.

[59] Sindagi V. A., Patel V. M. Generating high-quality crowd density maps using contextual pyramid cnns.

[60] Ranjan V., Le H., Hoai M. (2018). Iterative Crowd counting. *Proceedings of the European Conference On Computer Vision (ECCV)*.

[61] Shi M., Yang Z., Xu C. (2018). *Perspective-aware CNN for Crowd counting*.

[62] Chen X., Bin Y., Sang N., Changxin G. Scale Pyramid Network for Crowd counting.

[63] Luo Y., Pan J., Fan S., Zeyu D., Guanghua Z. (2020). Retinal image classification by self-supervised fuzzy clustering network. *IEEE Access*.

[64] Zhang A., Yue L., Shen J., Fan Z., Xiantong Z. Attentional neural fields for crowd counting.

[65] Wang B., Liu H., Samaras D. (2020). Distribution matching for crowd counting. *Advances in Neural Information Processing Systems*.

[66] Oh M. h, Olsen P., Ramamurthy K. N. (2020). Crowd counting with decomposed uncertainty. *Proceedings of the AAAI Conference on Artificial Intelligence*.

[67] He Y., Carass A., Zuo L., Blake E., Jerry L. (2020). Self Domain Adapted network. *International Conference On Medical Image Computing And Computer-Assisted Intervention*.

[68] Wang C., Song Q., Zhang B. (2021). Uniformity in heterogeneity: diving deep into count interval partition for crowd counting. *Proceedings of the IEEE/CVF International Conference on Computer Vision*.

[69] Ochs M., Kretz A., Mester R. (2019). Semantically Guided Depth Estimation network. *German Conference on Pattern Recognition*.

[70] Wan J., Liu Z., Chan A. B. A Generalized Loss Function for Crowd Counting and localization.

[71] Xu Y., Zhong Z., Lian D., Jing L., Zhengxin L., Xinxing X. Crowd counting with partial annotations in an image.

[72] Khan S. D., Salih Y., Zafar B., Noorwali A., Abdulfattah N. (2021). A deep-fusion network for crowd counting in high-density crowded scenes. *International Journal of Computational Intelligence Systems*.

[73] Khan S. D., Basalamah S. (2021). Sparse to dense scale prediction for crowd couting in high density crowds. *Arabian Journal for Science and Engineering*.

[74] Basalamah S., Khan S. D., Ullah H. (2019). Scale driven convolutional neural network model for people counting and localization in crowd scenes. *IEEE Access*.

